# PSMA-Targeted Theranostics in Glioblastoma Multiforme: Current Evidence and Future Perspectives

**DOI:** 10.3390/ijms27167112

**Published:** 2026-08-08

**Authors:** Francesca Russo, Maria Ricci, Habibollah Dadgar, Laura Travascio, Maria Silvia De Feo, Gabriele Brunotti, Sharjeel Usmani, Hossein Arabi, Farah M. Anwar, Aysar Najeh Khalaf, Luca Filippi, Andrea Cimini

**Affiliations:** 1 Nuclear Medicine Unit, St. Salvatore Hospital, 67100 L’Aquila, Italy; frarusso991@gmail.com (F.R.); gabriele.brunotti@gmail.com (G.B.); 2 Nuclear Medicine Unit, Cardarelli Hospital, 86100 Campobasso, Italy; maria.ricci28@gmail.com; 3 Cancer Research Center, RAZAVI Hospital, Imam Reza International University, Mashhad 9198613636, Iran; reza.lt.dadgar@gmail.com; 4 Nuclear Medicine Unit, Spirito Santo Hospital, 65124 Pescara, Italy; lauratravascio.lt@gmail.com; 5 Department of Radiological Sciences, Oncology and Anatomo-Pathology, Sapienza, University of Rome, 00161 Rome, Italy; mariasilvia.defeo@uniroma1.it; 6 Sultan Qaboos Comprehensive Care and Research Centre, University Medical City, Muscat 5661, Oman; dr_shajji@yahoo.com; 7 Division of Nuclear Medicine and Molecular Imaging, Department of Medical Imaging, Geneva University Hospital, CH-1211 Geneva, Switzerland; hossein.arabi@unige.ch; 8 Nuclear Medicine Department, Warith International Cancer Institute, Karbala 56001, Iraq; f.anwar@warith-ici.net (F.M.A.); aysar.najeh@whf.orq.iq (A.N.K.); 9 Department of Biomedicine and Prevention, University of Rome ‘Tor Vergata’, Via Montpellier 1, 00133 Rome, Italy; luca.filippi@uniroma2.it; 10 Nuclear Medicine Research Unit, IRCCS San Raffaele Roma, Via della Pisana 235, 00163 Rome, Italy

**Keywords:** PSMA, theragnostics, glioblastoma multiforme, nuclear medicine, radioligand therapy, PET, SPECT

## Abstract

Glioblastoma multiforme (GBM) is the most common brain tumor in the adult population, and it is characterized by poor prognosis; thus, new treatment strategies are strongly needed. Precision medicine is a rapidly growing field in oncology that identifies specific molecular features of tumor cells and allows theranostic strategies (combining diagnosis and therapy). In nuclear medicine, prostate-specific membrane antigen (PSMA) represents an interesting molecular target in theranostics, considering its involvement in the neoangiogenesis of many kinds of tumors, including GBM. In this narrative review, we performed a web-based literature search to explore the current evidence regarding PSMA-targeted nuclear medicine theranostics in GBM, highlighting diagnostic applications, therapeutic potential, and future clinical perspectives.

## 1. Introduction

### 1.1. Glioblastoma Multiforme: Background and Potential Theranostic Application Targeting PSMA

Glioblastoma multiforme (GBM) is the most common brain tumor in adults. It is a World Health Organization (WHO) grade IV glioma, and it is characterized by rapid growth and poor prognosis (median survival 12–15 months) [1]. Moreover, it is well known that the high cellular heterogeneity and the blood–brain barrier (BBB) structure give survival advantages to GBM cells [2]. Current treatment management of GBM includes a combined approach of surgery, radiotherapy and chemotherapy with temozolomide that remains insufficient to overcome the high aggressiveness of GBM cells; therefore, new weapons and treatment strategies are strongly needed.

Precision medicine is a rapidly growing field in oncology, identifying specific tumor molecular features and tailoring individual treatment to patients [3]. The importance of effective targeted therapies has been underlined in the literature [3,4], and several nuclear medicine techniques may assess specific molecular targets for diagnostic and therapeutic purposes with the use of radiopharmaceuticals, allowing theranostic strategies. Nuclear medicine imaging techniques, such as single-photon emission computed tomography (SPECT) or Positron Emission Tomography (PET)—which are commonly integrated with computed tomography (CT) or magnetic resonance imaging (MRI) in hybrid imaging platforms—enable the non-invasive assessment of specific biological targets, thereby supporting patient selection for targeted therapies and facilitating theranostic approaches [3]. In this context, a theranostic approach is based on the use of radiopharmaceuticals targeting the same molecular structure for both diagnostic imaging and radioligand therapy (RLT), allowing a personalized approach to cancer management.

It is well known that GBM is a highly vascularized tumor, and its aggressive behavior is closely associated with tumor angiogenesis [5]. In recent years, GBM endothelium has attracted considerable attention because, unlike the vasculature of many other brain tumors, it is not fully protected by the BBB and is therefore more exposed to circulating factors. Moreover, alterations in endothelial protein expression play a pivotal role in tumor neoangiogenesis, providing potential targets for the development of novel therapeutic strategies.

In this scenario, prostate-specific membrane antigen (PSMA) represents a potential theranostic target in consideration of its involvement in tumoral neoangiogenesis, and it has gained attention in the field of nuclear medicine theranostics [4]. PSMA is a type II transmembrane glycoprotein encoded by the gene FOLH1, and it plays an important role in neoangiogenesis of many kinds of tumors, including GBM [6]. Despite its name, this protein is not specific for prostate cancer, and it has been shown to be expressed in endothelial cells of neovasculature from benign and malignant conditions [7,8], but not in normal endothelium [9,10].

Regarding applications in oncology, the usefulness of theragnostic approaches targeting PSMA in metastatic prostate cancer is well-documented. Many PSMA-targeted radiopharmaceuticals are used in clinical practice for detection, staging, recurrence evaluation, and RLT in prostate cancer [11]. Moreover, potential and innovative theragnostic strategies have been described in other tumors [12]. In this context, evidence highlights the potential theranostic application targeting PSMA in GBM as well, paving the way for new imaging and treatment strategies [4,12].

### 1.2. Molecular and Biological Basis of PSMA in Glioblastoma Multiforme

As outlined above, PSMA has emerged as a promising target in GBM owing to its selective expression within tumor-associated neovasculature and its potential involvement in angiogenic processes. Understanding the biological role of PSMA in GBM is essential for evaluating its suitability as a target for nuclear medicine imaging and RLT. Recent studies showed that normal cerebral parenchyma expresses little or no PSMA either in brain cells or vessels, so its expression is exclusive to neovasculature in patients affected with GBM [10,13] and correlates negatively with survival [14].

Although the precise mechanisms underlying the role of PSMA in tumor angiogenesis have not yet been fully elucidated, evidence suggests that PSMA contributes to angiogenesis through several interconnected molecular pathways, including integrin-mediated signaling, extracellular matrix remodeling, and modulation of local folic acid metabolism [15,16]. In GBM, tumor neovasculature plays a key role in disease progression, and many studies demonstrated PSMA expression in the vessels of this malignancy to varying degrees [6,13], with moderate-to-high intensity, also with differences in initial diagnosis and recurrences. Several hypotheses have been proposed for the explanation of heterogeneity in PSMA expression, including differences in transcriptomic profile of FOLH1, but the mechanism remains unknown [17].

Several studies have demonstrated increased PSMA expression in the endothelial cells of high-grade gliomas, with higher levels associated with tumor aggressiveness and poorer prognosis [6,18,19,20]. Experimental evidence further suggests that PSMA may actively participate in GBM angiogenesis through interaction with integrin β4 (ITGB4), leading to activation of the nuclear factor kappa-light-chain-enhancer of activated B cells (NF-kB) signaling pathway [18,21]; in turn, activation of the ITGB4/NF-kb axis has been shown to upregulate PSMA expression in endothelial cells, suggesting the existence of a positive regulatory feedback loop that may promote endothelial tube formation and tumor neoangiogenesis [17,22,23]. Moreover, many studies have shown that abnormal activation of the NF-kB can cause an increase in the concentration of vascular endothelial growth factor (VEGF), another important target in tumor microvasculature, causing invasion of cancer cells [24].

PSMA seems to be independent of VEGF [25]. The level of VEGF expression correlates with glioma grade, and it is thought to be the main promoter of angiogenesis in glioblastoma [26], so potential interaction between PSMA and VEGF warrants further investigation, given that they are both possible targets in these malignancies. However, the relationship between PSMA and VEGF expression in GBM remains under investigation, and further evidence is expected from ongoing clinical trials (https://clinicaltrials.gov/study/NCT07052877?cond=Glioblastoma%20Multiforme&term=PSMA&viewType=Card&page=1&rank=1, accessed on 26 July 2026).

### 1.3. Scope of the Review

The aim of this narrative review is to provide a comprehensive overview of the current evidence on PSMA-targeted theranostic approaches in GBM, with particular emphasis on molecular imaging in nuclear medicine, radioligand therapy, current limitations, and future research directions. Furthermore, it aims to identify current knowledge gaps and highlight the potential of PSMA-targeted strategies as a promising field for future investigation.

## 2. Methods

This narrative review was conducted through a literature search aimed at identifying published evidence on PSMA-targeted imaging and RLT in GBM.

A comprehensive literature search was performed in PubMed/MEDLINE and Scopus. The final search was conducted on 30 June 2026, and the following keywords were used:

(“glioblastoma” OR “glioblastoma multiforme” OR “high-grade glioma”) AND (“PSMA” OR “prostate-specific membrane antigen”) AND (“PET” OR “SPECT” OR “molecular imaging” OR “radioligand therapy” OR “theranostics”)

Original articles, clinical studies, case reports, and relevant preclinical investigations published in English were considered for inclusion. Additional publications providing background information on GBM, molecular imaging in nuclear medicine, RLT, or theranostic approaches were also included when considered relevant to the aims of this review. Editorials, conference abstracts without sufficient data, and duplicate publications were excluded.

Two authors (F.R. and A.C.) screened the titles and abstracts of the retrieved records, followed by full-text evaluation of potentially eligible publications.

**Figure 1:**
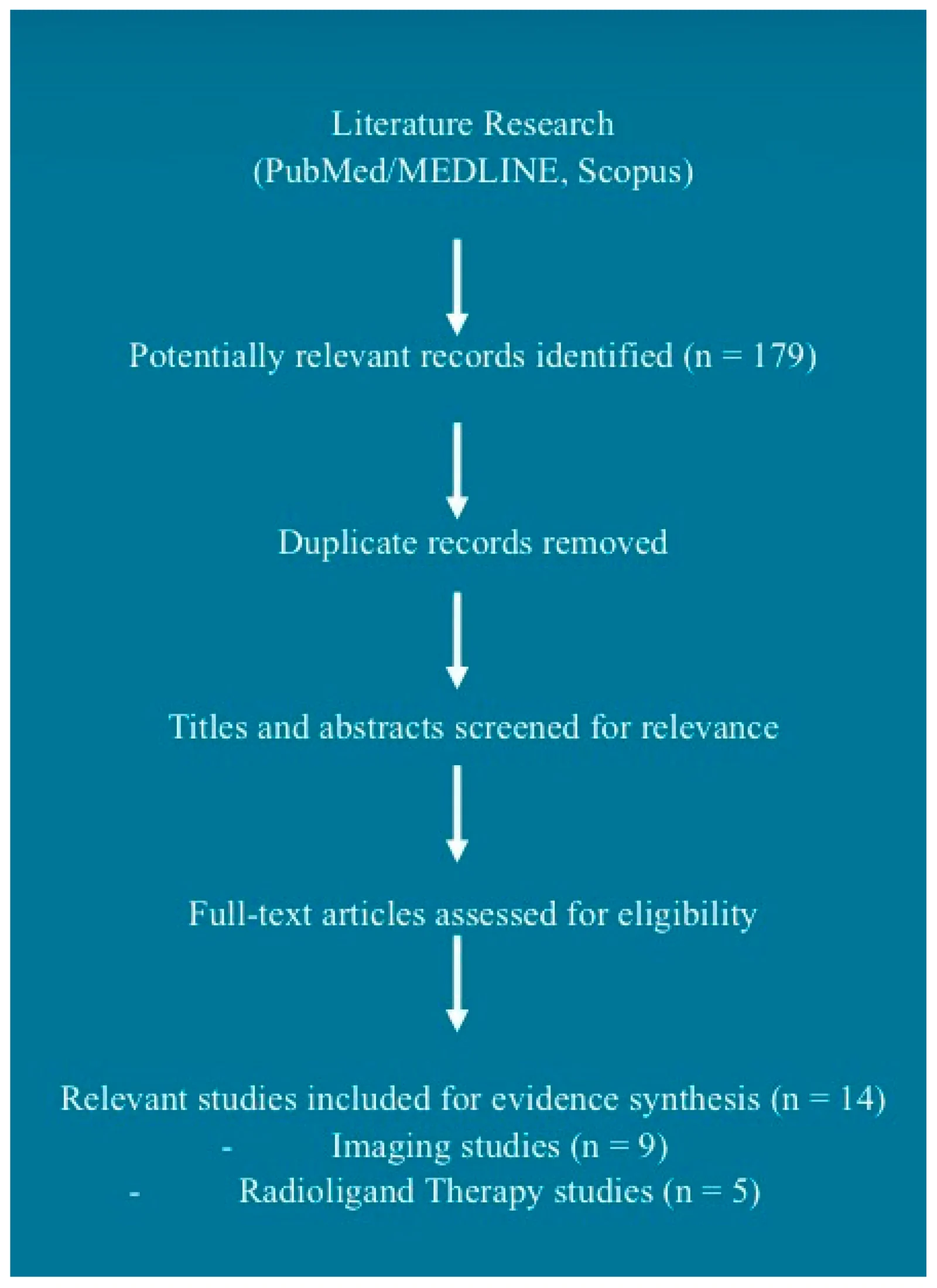
Schematic overview of the literature identification and study selection process adopted for this narrative review.

Given the narrative nature of the review and the limited number of available publications, no formal quality assessment or meta-analysis was performed. The selected publications were qualitatively analyzed to summarize current evidence regarding diagnostic imaging, RLT, and future perspectives of PSMA-targeted theranostics in GBM. A schematic overview of the literature identification and study selection process adopted for this narrative review is provided in Figure 1.

## 3. PSMA and Nuclear Medicine Imaging in Glioblastoma Multiforme

Although MRI remains the gold standard for the diagnosis and follow-up of glioblastoma, PET imaging provides complementary functional information and has emerged as a promising tool for the characterization of brain tumors. In this context, PET imaging with amino acid tracers such as [^11^C-methyl]-methionine ([^11^C]MET), *O*-(2-[^18^F]fluoroethyl)-L-tyrosine ([^18^F]FET) and 3,4-dihydroxy-6-[^18^F]fluoro-L-phenylalanine ([^18^F]DOPA) may contribute to glioma grading, detection of multifocal disease, and differentiation between tumor recurrence and treatment-related changes [27].

**Figure 2:**
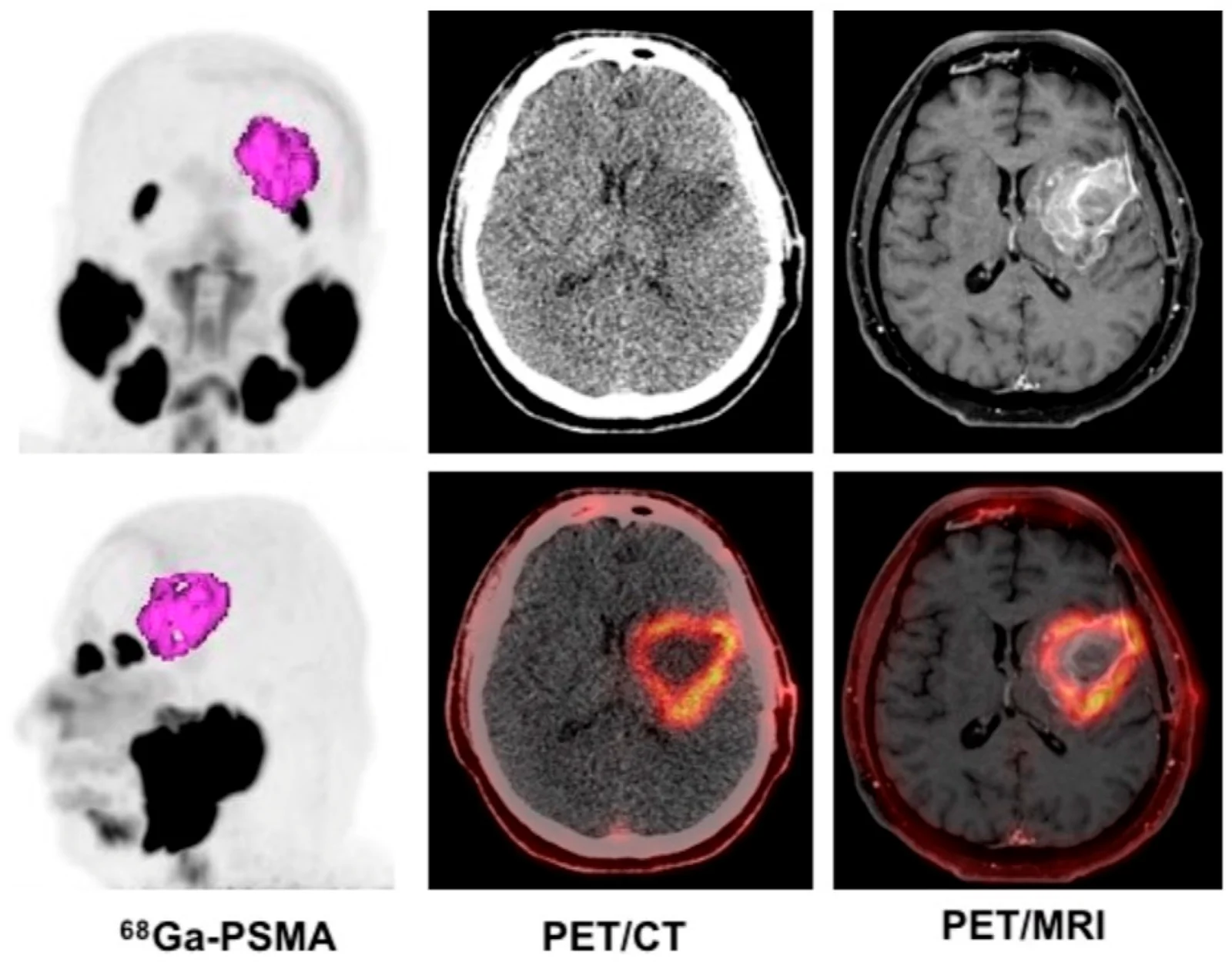
A 44-year-old male patient with glioblastoma multiforme (IDH-wildtype), treated with craniotomy: ^68^Ga-PSMA positron emission tomography (PET)/computed tomography (CT) and PET/magnetic resonance imaging (MRI) axial images show a lesion with abnormal PSMA expression in the left fronto-temporal region, with maximum standardized uptake value (SUVmax) 4.6 and metabolic tumor volume (MTV) 27.9 cm^3^ at PET/CT images. Findings are consistent with a significant active residual tumor characterized by neovascularization. Written informed consent has been obtained from the patient to publish this figure.

As we highlighted before, the rationale for PSMA-targeted imaging in GBM derives from the expression of PSMA within tumor-associated neovasculature (Figure 2). In this regard, several studies have demonstrated the feasibility and the promising role of non-invasive assessment of PSMA expression in both newly diagnosed and recurrent GBM using PSMA-targeted PET radiopharmaceuticals [8,11,19,28,29,30]. Furthermore, interindividual variability and temporal changes in PSMA expression between primary and recurrent lesions have been reported, reflecting the biological heterogeneity of the disease [8,18].

An important advantage of PSMA PET imaging in brain tumors is the very low physiological uptake of PSMA-targeted tracers in normal cerebral tissues, resulting in excellent tumor-to-background ratios (TBRs) and facilitating image interpretation [29]. In this regard, Kunikowska et al. reported a median TBR value of 96.7 in a study involving GBM patients who underwent [^68^Ga] Ga-PSMA-11 PET/CT [31]. In addition, tracer uptake has been shown to correlate with tumor grade and proliferation indices such as Ki-67, supporting the association between PSMA expression and tumor aggressiveness [32]; these characteristics have generated interest in the potential role of PSMA PET for differentiating high-grade from low-grade gliomas [32].

Most available evidence has been obtained using radiotracers labeled with Gallium-68, particularly [^68^Ga] Ga-PSMA-11, which remains the most extensively investigated compound in neuro-oncology [31]. Nevertheless, fluorinated tracers such as ^18^F-Piflufolastat (also known as [^18^F] F-DCFPYL) have also shown promising results [30]. Biodistribution and time–activity curves appear largely comparable between gallium- and fluorine-labeled compounds, especially when comparing ^18^F-Piflufolastat and [^68^Ga]Ga-PSMA-11 [30], but the use of ^18^F-labeled tracers may offer practical advantages related to production and distribution [33]; however, comparative studies specifically addressing GBM are currently lacking.

An important application of PSMA PET concerns the evaluation of recurrent disease. In fact, preliminary evidence suggests that PSMA-targeted imaging may help distinguish tumor recurrence from radionecrosis, a clinically relevant challenge in the follow-up of GBM patients. In this setting, PSMA PET has demonstrated favorable TBR values and, in some reports, has shown potential advantages over amino acid tracers such as ^18^F-FET [19,28,34]. However, larger prospective studies are required to validate these findings. In this regard, PET imaging with radiolabeled PSMA minibody such as ^89^Zr-Df-IAB2M may have a potential value in distinguishing recurrence from radionecrosis, as demonstrated in a previous study by Matsuda et al. evaluating 83 brain tumors (including 41 GBM) [35].

PSMA expression in PET imaging may also provide prognostic information. In 2021, Holzgreve et al. demonstrated, in a group of 16 patients with GBM, that high vascular PSMA expression, particularly at recurrence, is associated with shorter survival and poorer clinical outcomes [14]. Moreover, increasing PSMA expression over time appears to correlate with disease progression, suggesting a possible role as a prognostic biomarker [14].

Beyond diagnosis, PSMA PET may play a role in treatment planning. Several investigations have explored the integration of PSMA PET with MRI for target volume delineation prior to radiotherapy, reporting improved visualization of tumor margins, particularly in non-enhancing lesions [29,36]. Nevertheless, discrepancies between biological tumor volumes defined by PSMA PET and gross tumor volumes identified on MRI have been observed, suggesting that PSMA PET should currently be considered complementary rather than alternative to conventional imaging modalities.

It is important to underline that PET imaging may also represent a crucial step in patient selection for PSMA-targeted RLT. A correlation between PET uptake and endothelial PSMA expression assessed by immunohistochemistry has been demonstrated, supporting the use of PSMA PET as a biomarker for identifying patients who may benefit from therapeutic approaches using radiolabeled PSMA ligands [18]. However, compared with prostate cancer, GBM generally exhibits lower standardized uptake value (SUV) and TBR values, limiting the proportion of patients who may be considered potential candidates for PSMA-directed RLT [13,16,37,38].

In comparison to PET, SPECT presents lower spatial resolution and lower image quality [39,40]; however, it remains a clinically useful imaging modality and may serve as a practical alternative when PET is not available. In this context, Ghaedian et al. evaluated 31 GBM lesions using SPECT/CT with the PSMA-targeted radiopharmaceutical [^99m^Tc]-Tc-HYNIC-PSMA-11. The authors demonstrated the promising reliability of this imaging technique for the detection of GBM lesions, especially using quantitative TBR analysis [41].

Overall, despite the heterogeneity of the available studies, the current evidence supports the potential clinical value of PSMA-targeted imaging in GBM. However, larger prospective studies with standardized imaging protocols and semiquantitative assessment are still required before its clinical role can be fully established.

## 4. PSMA and Radioligand Therapy in Glioblastoma Multiforme

The encouraging preliminary imaging findings obtained with PSMA-targeted PET have naturally stimulated interest in the development of PSMA-RLT for GBM; however, biological and technical challenges currently limit the clinical application of this strategy.

A major limitation is represented by the predominantly vascular expression of PSMA in GBM. Unlike prostate cancer cells, GBM cells generally do not exhibit significant internalization of PSMA-targeted radioligands, resulting in relatively short tracer retention times within tumor tissue [42]. This aspect may reduce the therapeutic efficacy of RLT, particularly when using α-emitting radionuclides, whose short path length (40–100 μm) requires prolonged retention at the target site to maximize radiation-induced cellular damage [42]. Despite these limitations, the available preliminary clinical experience has stimulated further interest in the development of PSMA-targeted radioligand therapy for selected patients with recurrent high-grade gliomas [43]. Among the available radionuclides, β-emitting agents such as lutetium-177 are generally considered more suitable because of their longer tissue penetration range (0.05–12 mm), which may allow a crossfire effect capable of irradiating adjacent tumor cells even in the absence of direct tracer uptake [44]. Kumar et al. highlighted the potential feasibility of PSMA-RLT in a 37-year-old patient with GBM, with a significant reduction in lesion size (demonstrated on MRI) after 3 cycles of [^177^Lu] Lu-PSMA-617 (and a significant clinical improvement in the patient’s symptoms reported) [45]. A recent case report showed the efficacy of ^177^Lu-PSMA RLT in a 42-year-old patient with GBM, with excellent response to treatment (assessed both with MRI and [^99m^Tc]-Tc-HYNIC-PSMA-11 scintigraphy) after 6 doses of [^177^Lu] Lu-PSMA-617 [46]. Furthermore, a recent study by Ghaedian et al. evaluated the feasibility of ^177^Lu-PSMA therapy in 10 patients with non-responsive or progressive GBM receiving standard therapies: the authors demonstrated safety (no significant hematologic or renal toxicity was reported) and effectiveness of RLT in these patients, with 6-month and 1-year survival rates of 100% and 80%, respectively, after the first dose of ^177^Lu-PSMA therapy [47].

In contrast, Graef et al. evaluated intratherapeutic dosimetry following [^177^Lu] Lu-PSMA-RLT and reported a median absorbed tumor dose of only 0.56 Gy, concluding that the therapeutic efficacy of this approach in high-grade gliomas remains questionable despite measurable tracer uptake [48]: these findings appear to contrast with the aforementioned case reports and small case series [45,46,47]. This apparent discrepancy may reflect the marked biological heterogeneity of high-grade gliomas, particularly GBM, including variability in PSMA expression and radioligand uptake and retention, although the relative contribution of these factors remains to be established. Moreover, the currently available clinical evidence remains limited and is based almost exclusively on individual cases and small patient cohorts as described before [45,46,47,48].

Alternative administration strategies are also being investigated to improve radioligand delivery. Intra-arterial delivery has attracted attention because it may increase local radioligand concentration within the tumor while maintaining an acceptable safety profile [49,50,51,52]. Preliminary studies suggest that this approach could improve tumor dosimetry and optimize therapeutic efficacy, especially when combined with pre-treatment PET imaging and individualized dosimetric assessment. In this regard, Pruis et al. evaluated 10 brain tumor patients (including 4 patients with GBM) with [^68^Ga]Ga-PSMA-11 PET/MRI, administering the radiopharmaceutical intravenously and intra-arterially; intra-arterial administration led to a fifteen-fold higher radioligand uptake at the lesion site (with no treatment-related toxicities reported), suggesting that this approach may also have potential implications for PSMA-RLT by enhancing tumor delivery and potentially improving therapeutic efficacy [51].

Another important consideration concerns treatment safety. As PSMA is physiologically expressed in several normal organs, including salivary and lacrimal glands, kidneys, liver, spleen, intestines, and urinary bladder, careful dosimetric evaluation is required to maximize tumor irradiation while limiting radiation exposure to healthy tissues [35]; consequently, investigations should also be focused on defining optimal eligibility criteria, including minimum TBR thresholds for treatment selection.

Collectively, the available therapeutic studies support the feasibility of PSMA-RLT in selected GBM patients. However, the current evidence remains limited to case reports and small series, preventing robust conclusions regarding this approach.

An overview of the most relevant research articles concerning PSMA-targeted nuclear medicine imaging in GBM cited in the manuscript is reported in Table 1. An overview of the publications regarding PSMA-targeted nuclear medicine therapy in GBM cited in the manuscript is reported in Table 2. Given the limited number of available studies and the possibility of multiple publications originating from the same institution, potential overlap between patient cohorts cannot be excluded. The available information reported in the original publications was carefully evaluated; however, it was not possible to determine with certainty whether overlapping patient populations were present.

## 5. Discussion

The available evidence suggests that PSMA-targeted theranostics may represent a promising strategy for the management of GBM. Although PSMA represents one of the most investigated vascular targets in GBM, several alternative molecular targets are currently under evaluation for theranostic applications, such as C-X-C chemokine receptor type 4 (CXCR-4) [53,54], fibroblast activation protein (FAP) [55,56], and epidermal growth factor receptor (EGFR) [56]. Compared to these molecular targets, PSMA benefits from the availability of clinically established radiopharmaceuticals and extensive experience gained in oncology (especially in prostate cancer).

Overall, PSMA-targeted RLT represents a promising therapeutic approach for GBM, although several challenges currently limit its clinical implementation, and while preliminary evidence has demonstrated its feasibility in selected patients, further studies are needed before this approach can be translated into routine clinical practice. As we described before, PSMA-targeted nuclear medicine imaging in GBM (with PET or SPECT) may have potential applications in tumor characterization, recurrence assessment, radiotherapy planning, and patient selection for RLT; as regards the last point, patient selection remains challenging, and the integrity of the BBB and the heterogeneous permeability of the blood–tumor barrier may critically influence the delivery of PSMA-targeted radiopharmaceuticals. In this context, intra-arterial radiopharmaceutical administration may overcome limitations, enhancing tumor delivery and optimizing RLT efficacy as well [57,58]. Future research in this context is needed, but the role of intra-arterial radiopharmaceutical administration in patient selection for PSMA-RLT in GBM seems to be promising. In addition, although β-emitting radionuclides currently appear more suitable for PSMA-RLT in GBM, α-emitting radionuclides should not be completely excluded. In this regard, intra-arterial administration may increase the potential efficacy of α-particle therapy in selected patients, improving radioligand retention [59]. More broadly, the potential role of α-emitting radionuclides and other emerging theranostic strategies in GBM has recently been comprehensively reviewed by Roncali et al. [60].

Dosimetry may become a key determinant of treatment success in GBM. Because PSMA expression is confined mainly to tumor neovasculature, absorbed tumor doses may differ substantially from those achieved in metastatic prostate cancer. Individualized dosimetry is increasingly recognized as a cornerstone of RLT, allowing optimization of tumor absorbed dose while minimizing radiation exposure to organs at risk and supporting a more personalized treatment approach [61,62]. Therefore, future studies should investigate individualized dosimetric approaches in PSMA-RLT for GBM patients, establishing dose thresholds associated with clinical response.

An additional aspect deserving consideration is the choice of the PSMA-targeting ligand. Brighi et al. reported that [^68^Ga] Ga-PSMA-617 shows lower nonspecific renal uptake than [^68^Ga] Ga-PSMA-11 [63], the radiopharmaceutical employed in the study by Pruis et al. [51]. This observation may be clinically relevant in this context, as radiation exposure to the kidneys represents one of the principal limitations of PSMA-targeted RLT [64]. In addition, [^68^Ga] Ga-PSMA-617 has been shown to accumulate not only within lesions but also in peritumoral regions characterized by early neoangiogenic activity, where BBB disruption is not yet evident, suggesting a potential advantage for theranostic applications. By contrast, uptake of [^68^Ga] Ga-PSMA-11 appears to be more closely associated with regions of BBB impairment, which may reduce its ability to target tumor cells [64]. Overall, optimization of ligand selection may represent a key factor for PSMA-targeted theranostic approaches in GBM.

It is well-known that artificial intelligence (AI) methods may provide reliable assessments of tumor burden on PSMA PET in prostate cancer, allowing survival prediction after RLT as well [65]. As a future perspective, similar approaches may be applied in GBM, where AI-based analysis of PSMA PET images may improve lesion characterization, facilitate automated quantification of tracer uptake, and support patient selection for PSMA-targeted therapies. Furthermore, the integration of PET-derived radiomic features with clinical, histopathological, and molecular data may enable the development of predictive models for treatment response and prognosis, contributing to a more personalized theranostic approach. Collectively, these emerging technologies may contribute to a more personalized theranostic approach once validated in prospective clinical studies.

Despite the encouraging results reported, the current evidence supporting PSMA-targeted imaging and RLT in GBM remains limited. Most available data derive from case reports and small single-center cohorts, resulting in substantial heterogeneity in patient populations, imaging protocols, and radiopharmaceuticals. Larger prospective multicenter studies are required to validate current findings. Moreover, important insights may be gained from the results of ongoing clinical trials (https://clinicaltrials.gov/study/NCT07223034?cond=Glioblastoma%20Multiforme&term=PSMA&viewType=Card&page=1&rank=8, accessed on 26 July 2026) to define the precise role of PSMA-targeted theranostics in clinical practice.

### Current Limitations and Future Perspectives

Overall, the currently available evidence indicates that PSMA-targeted theranostics in GBM has reached a proof-of-concept stage; nevertheless, several challenges still prevent its routine implementation. Based on the available literature, three major priorities emerge for the future development of this field. First, larger prospective multicenter studies are required to validate the promising diagnostic performance of PSMA-targeted imaging and to establish standardized quantitative criteria for patient selection. Second, optimization of therapeutic strategies should focus on individualized dosimetry, improved radioligand delivery, and identification of the most appropriate PSMA-targeting ligand. Finally, emerging approaches, including intra-arterial administration, α-emitting radionuclides, and AI-based quantitative image analysis, may further improve treatment personalization, although their clinical value remains to be demonstrated in prospective clinical studies.

## 6. Conclusions

PSMA-targeted theranostics in GBM represents an emerging and promising field of research, with encouraging results reported for PSMA-targeted imaging, whereas therapeutic applications have so far been limited to a small number of patients. However, current evidence remains preliminary, as it is based predominantly on case reports and small retrospective single-center studies, without prospective controlled trials. Larger prospective studies are needed to clarify patient selection, optimize dosimetry, and determine whether PSMA-RLT can provide meaningful survival benefits in this highly aggressive disease. The evolution of PSMA-targeted theranostics in GBM will likely depend on future research, paving the way toward a truly personalized theranostic approach.

## Figures and Tables

**Table 1:** Summary of the most relevant studies on PSMA-targeted nuclear medicine imaging in GBM cited in the paper.

Authors	Year	Study Population	Study Overview	Main Findings	Semiquantitative Imaging Parameters
Holzgreve et al. [14]	2021	16 de novo and recurrent Glioblastoma multiforme (GBM)	Patients underwent [^68^Ga]-PSMA-11 Positron emission tomography (PET)/Computed Tomography (CT) at recurrence.	PSMA PET scan showed high tumor-to-background ratio (TBR) contrast ma but low intra-tumoral uptake of the radiopharmaceutical	Maximal TBR 21.6 in one patient reported
Truckenmueller et al. [18]	2022	20 patients with relapsing high-grade glioma (17 GBM)	Patients underwent [^68^Ga] Ga-PSMA PET/Magnetic Resonance Imaging (MRI) to evaluate eligibility for [^177^Lu] Lu-PSMA therapy	Usefulness of PSMA PET imaging in patient selection for radioligand therapy (RLT). Three patients were eligible for [^177^Lu] Lu-PSMA therapy.	Patients eligible for RLT showed maximum TBR > 1 and median maximum standard uptake value (SUVmax) 8
Sasikumar et al. [28]	2017	5 GBM Patients	Patients underwent [^18^F] F-FDG and [^68^Ga] Ga PSMA-11 PET/CT	Abnormal tracer uptake in 4 out of 5 GBM patients. No uptake of [^68^Ga] Ga-PSMA-11 in normal brain parenchyma results, with high TBR values: excellent visualization of active disease.	The 4 GBM patients with abnormal [^68^Ga] Ga-PSMA-11 uptake demonstrated TBR values of 12.9, 4.07, 1.15, and 22.3, respectively.
Şahin M et al. [29]	2022	10 GBM surgically treated	Radiotherapy planning Computed Tomography (CT) images were fused separately with pre-operative Magnetic Resonance Imaging (MRI) and PET/MRI images of 10 GBM patients.	[^68^Ga] Ga-PSMA PET may clearly define tumor borders in GBM patients	Median SUVmax value reported was 9.45 (5.58–39.58)
Kunikowska et al. [31]	2020	15 GBM patients	All patients underwent MRI and [^68^Ga] Ga-PSMA-11 PET/CT	Promising role of [^68^Ga] Ga-PSMA-11 PET/CT in the assessment of recurrence in GBM patients.	Median lesion SUVmax was 6.5 (range 2.1–14.3). Excellent median TBR value reported (96.7).
Akgun et al. [32]	2020	35 patients with glioma (13 GBM patients)	All patients underwent [^68^Ga] Ga-PSMA PET/MRI	Promising role of [^68^Ga] Ga-PSMA PET/MRI in differentiating Grade IV glial tumors from other grades. Significant correlation between Ki-67 and SUVs.	Mean SUVmax reported was 3 ± 3.6 (0.2–39.58)
Matsuda et al. [35]	2018	41 GBM	Evaluation of PSMA expression in brain tumors using PSMA antibody/minibody	PET imaging with radiolabeled PSMA minibody (^89^Zr-Df-IAB2M) may be useful in distinguishing recurrence from radionecrosis.	Not reported
Ghaedian et al. [41]	2026	31 lesions with suspected GBM recurrence	Evaluation of diagnostic accuracy of single photon emission computed tomography (SPECT)/CT with [^99m^Tc]-Tc-HYNIC-PSMA-11 for the detection of GBM lesions.	Promising reliability of SPECT/CT with [^99m^Tc]-Tc-HYNIC-PSMA-11 for the detection of GBM lesions. Visual assessment demonstrated a sensitivity of 83% and specificity of 92%.	Quantitative SPECT/CT analysis revealed a mean TBR of 13.08 ± 12.09.
Pruis et al. [51]	2024	10 patients with brain tumors (4 GBM)	Evaluation of [^68^Ga] Ga-PSMA-11 tumor uptake with intra-arterial administration	Intra-arterial administration led to a fifteen-fold higher [^68^Ga] Ga-PSMA-11 uptake at the lesion site compared to intravenous administration: potential implications for PSMA-RLT by enhancing tumor delivery.	SUVmax median with Intra-arterial administration at lesion site: 142.8. SUVmax median with intravenous administration: 10.5.

**Table 2:** Summary of the studies on PSMA-targeted nuclear medicine therapy in GBM cited in the paper.

Authors	Year	Study Population	Study Overview	Main Findings	Adverse Events
Kunikowska et al. [43]	2020	1 patient with recurrent GBM	Dosimetry study, [^177^Lu] Lu-PSMA-617 treatment in a GBM patient.	Post-therapy images demonstrated increased uptake of [^177^Lu] Lu-PSMA-617 in the tumor lesion during the first 24 h, with a subsequent decreased accumulation (up to 14 days). The absorbed dose for the tumor was 14.07 Gy, kidney 0.14 Gy, liver 1.67 Gy, and for the whole body 0.49 Gy.	Not reported (NR)
Kumar et al. [45]	2020	1 patient with recurrent GBM	[^177^Lu] Lu-PSMA-617 radioligand therapy (RLT) in a GBM patient	Exploratory [^68^Ga] Ga-PSMA-11 PET/CT demonstrated intense PSMA expression in the lesion. Significant reduction in lesion size after 3 cycles of [^177^Lu] Lu-PSMA-617 therapy.	No significant adverse events.
Ghaedian et al. [46]	2026	1 patient with recurrent GBM	[^177^Lu] Lu-PSMA-617 RLT in a GBM patient	6 doses of 100–150 mCi [^177^Lu] Lu-PSMA-617 RLT were administered: excellent response on both MRI and ^99m^Tc-HYNIC-PSMA-11 scintigraphy.	NR
Ghaedian et al. [47]	2026	10 patients with GBM	[^177^Lu] Lu-PSMA RLT in GBM patients	Safety and effectiveness of ^177^Lu-PSMA RLT in GBM patients (patients received 2–6 cycles of 100–150 mCi). 6-month and 1-year survival rates of 100% and 80%, respectively, after the first dose of ^177^Lu-PSMA therapy.	No significant adverse events. No significant hematologic or renal toxicity noted.
Graef et al. [48]	2023	3 patients with high-grade gliomas	[^177^Lu] Lu-PSMA RLT in high-grade gliomas	Median absorbed tumor dose was only 0.56 Gy: questionable therapeutic efficiency.	1 patient (with underlying myelodysplastic syndrome) showed thrombocytopenia.

## Data Availability

No new data were created or analyzed in this study. Data sharing is not applicable to this article.
